# Treatment of Burns and Scalds

**Published:** 1875-05

**Authors:** John Morris

**Affiliations:** Baltimore, in Sanitarian.


### ARTICLE VIII.

### *Treatment of Burns and Scalds.*

The first step is to remove the clothing from the patient. As rest is all-important, this should not be done by the old plan of taking it off piece by piece, but by removing it by a few skilful cuts with a knife or scissors. The patient should then be instantly wrapped in a blanket, or blankets, or large masses of cotton, if at hand, so as to create heat, and thus re-establish the circulation.

Patients frequently exhaust themselves by their outcries, and to guard against the depression of nervous force brought about by this cause, anæsthetics should at once be employed. Chloroform or ether should be administered in sufficient quantity to induce partial, or, if necessary, complete unconsciousness. If these agents are not at hand, large doses of opium should be given. This is all-important, as the patient must not be allowed to suffer if we wish to conserve the powers of life. The dressing should be made while the patient is in this state.

Carron oil is utterly useless, if not injurious. Of all the oils, linseed, in our opinion, is the worst, as it is the soonest to be absorbed by the atmosphere, and become dry. In cases of bad scalds of children, in which a large part of the body is involved, we know no dressing so good as a bran bed, that is, a bed of bran, in which the patient may lie, and be entirely covered with a thick investment of the same. This dressing has the advantage of not requiring change, for each day, as the moist particles fall off, they can be replaced with fresh bran without disturbing the patient. One of the severest cases of scald we ever met, recovered by this treatment.

A great deal of harm is done to patients by frequent dressings, and any method that obviates this is most desirable. Patients frequently are exposed for hours to the action of the air, suffering unnecessary pain, by the old and tedious process of dressing. The air itself does no injury, but the extreme hyperæsthesia of the skin produces a state of nervous tremor which leads to exhaustion. Any one who has seen a case of hydrophobia can readily understand this condition of skin hyperæsthesia.

In burns of the extremities, there is no immediate application so serviceable to relieve pain as hot or cold water, and, strange to say, they act equally well. If the appliances are at hand, the cold bath as practiced by Hebra is the best. Those who have visited his wards in Vienna, and seen his treatment of burns by a bed made of straps, in a cold bath, can bear witness to the successful and scientific character of this procedure. For small burns, warm water acts admirably.

We have said before, that anæsthetics should be employed in all burns of an extensive character, but, before their effect is allowed to pass off, applications should be made to produce anæsthesia of the parts affected. We have heretofore used for this purpose a solution of Labarraque's chloride of soda, of the strength of an ounce to a pint of water, adding two or three grains of morphia to the solution. This has generally given great relief to the patient—indeed, in a short time, destroying all the extreme sensibility. Carbolic acid has been highly recommended as a local anæsthetic, and it may be possible that a solution of it in water, in combination with morphia, might act still better.

After a free application of either of these solutions, the parts may be thickly covered with cotton batting. This helps to counterbalance the chilliness, and gives a comparative degree of comfort.

In superficial burns, of a limited extent, nothing is required but simple cold water dressing.

Brandy should not be administered whenever opium or ether can be obtained, as it remotely exercises a depressing influence. Strong hot coffee is the best drink that can possibly be given to counteract nervous exhaustion, or remedy the effects of shock. If brandy is given at all, it should be given with coffee. All earhy applications such as chalk, calaminaria, etc., should be avoided, as they are not only therapeutically inert, but may interfere with the process of restoration.

Local stimulation, such as the application of turpentine, or a solution of nitrate of silver, as practiced at St. Bartholomew Hospital, is no doubt proper treatment in the second stage of burns, but as this belongs more especially to the domain of surgery, we forbear to discuss it, as well as the treatment of the after consequences of burns, such as ulceration of the bowels, particularly of Peyer's glands, congestion of the lungs, cicatricial contractions, etc.

In conclusion, we will briefly sum up the recommendations before suggested:

1. Remove the clothing by cutting it from the body.
2. Wrap the patient in blankets.
3. If the pain be excessive, administer chloroform, ether, or large doses of opium, and let the necessary dressing be made while the patient is in a state of partial or total insensibility.
4. Produce anæsthesia of the burned or scalded parts by the application of a solution of carbolic acid and morphia. (This solution can be made in almond or olive oil.)
5. After this, wrap the patient in masses of cotton batting.
6. Avoid brandy, and give coffee as a stimulant.

If these simple rules be followed, much suffering may be alleviated, and many a life saved, which otherwise would be lost by the ignorance and mismanagement of attendants. —*Dr. John Morris, of Baltimore, in Sanitarian.*

